# Facile fabrication of selegiline-loaded alginate hydrogel for neuroprotection and functional recovery in a rat model of spinal cord injury through localized spinal delivery

**DOI:** 10.22038/ijbms.2025.81837.17706

**Published:** 2025

**Authors:** Ramin Abrishami, Ramtin Farhadi, Mehri Farhang Ranjbar, Seyed Hadi Aghili, Maryam Baeeri

**Affiliations:** 1 Research Center for Trauma in Police Operations, Directorate of Health, Rescue & Treatment, Police Headquarters, Tehran, Iran; 2 Department of Clinical Pharmacy, Faculty of Pharmacy, Tehran Medical Sciences, Islamic Azad University, Tehran, Iran; 3 Department of Toxicology and Pharmacology, Faculty of Pharmacy, Tehran University of Medical Sciences, Tehran, Iran; 4 Toxicology and Diseases Specialty Group, Pharmaceutical Sciences Research Center (PSRC), Tehran University of Medical Sciences, Tehran, Iran; 5 Department of Support and Services Management, Institute of Management and Organizational Resources, Policing Sciences and Social Studies; 6 Research Institute, Tehran, Iran; 7 Department of Medical-Surgical Nursing, School of Nursing and Midwifery, Tehran University of Medical Sciences, Tehran, Iran; 8 Neurosurgery Department, Imam Khomeini Hospital Complex, Tehran University of Medical Sciences, Tehran, Iran; 9 Valiasr Hospital, Department of Neurosurgery, Tehran, Iran

**Keywords:** Alginate hydrogel, Apoptosis, Functional recovery, Selegiline, Spinal cord Injury

## Abstract

**Objective(s):**

Spinal cord injury (SCI) is a highly disabling and fatal disorder with no effective treatment to date. Selegiline, a selective MAO-B inhibitor, has shown new neuroprotective and neurorescuing effects with various beneficial effects on neuron-associated disorders. These effects have triggered investigations into its impact on different neuron-associated disorders and SCI. Thus, in continuation of the previous studies, this study evaluates the local therapeutic effects of selegiline-loaded alginate hydrogel on SCI by analyzing apoptotic factors, histological factors, and improvements in locomotor function and neuropathic pain.

**Materials and Methods:**

Hydrogels were fabricated via cross-linking gelation method and characterized by FT-IR and SEM analysis. Selegiline release from hydrogels was evaluated by UV spectroscopy, and hydrogel biocompatibilities were verified through an MTT assay. Afterward, 36 rats were divided into six groups: sham, negative group, treated with empty hydrogel, and three selegiline-treated groups (2.5, 5, and 10 mg/kg). After 28 days, the locomotor activity, the expression of Bax and Bcl2 (apoptosis index), and GFAP changes in the lesion site were assessed using Basso, Beattie, and Bresnahan (BBB) scale, western blot technique, and immunohistochemical assay, respectively.

**Results:**

Hydrogel tests showed the suitability of hydrogels and sustained selegiline release from them. Rats treated with selegiline-loaded hydrogels showed significant locomotor improvement and reduced apoptosis indices in SCI-induced rats (*P*≤0.05). Additionally, GFAP immunohistochemistry analysis indicated notable histological improvements.

**Conclusion:**

Findings suggest that selegiline-loaded hydrogels can improve SCI through apoptosis inhibition and neurorescuing effects. Further clinical studies are warranted to validate these findings in human SCI.

## Introduction

Spinal cord injury (SCI) is a mortal and very disabling condition that results in various physical complaints, including impaired locomotor function, neuropathic pain, and eventually death (1). SCI is caused by different factors, of which trauma is the most common, and brings about many personal and societal burdens (2-4). According to the latest announcement by the World Health Organization (WHO), there are 250 to 500 thousand new cases of SCI in the world every year. Also, the latest studies revealed that there are 20 to 26 million patients with SCI globally, of which 90% are caused by traumatic incidents (5). Although finding an effective treatment for SCI is necessary due to its dangerous and disabling condition, some challenging problems make that a constant failure for researchers (6). The limited regeneration capacity of neuronal cells (7) and the progressively worsening nature of SCI have made reaching the appropriate clinical outcome unachievable (8). Up to today, methylprednisolone, with relatively small beneficial effects and serious side effects, is the only approved drug for SCI, which represents limited locomotor function improvements (9). There are serious adverse effects according to methylprednisolone administration, including infection, pneumonia, and hyperglycemia. Due to that, the FDA no longer recommends its use for the acute treatment of SCI, and it worsens when no other approved effective treatments are available for SCI that provide significant functional recovery (10, 11).

Selegiline is a selective inhibitor of monoamine oxidase-B (MAO-B) and has been used as an adjuvant medication in Parkinson’s disease since 1970 (12). Further investigations demonstrate its therapeutic potential in major depressive disorder and Alzheimer’s disease (13-15). The beneficial effects of selegiline on neuron-related diseases triggered more studies to investigate the effects of selegiline on the neurons more deeply. These further studies indicate that selegiline has various beneficial effects, such as anti-apoptotic (16), neuroprotective, and neurorescuing effects (17-19). Also, selegiline can increase the expression of neurotrophic genes (including BDNF, which is the most important one), similar to growth factors (20, 21). Tissue repair is another effect of selegiline, which is caused by increased gliosis and reduced astrocytes (22). According to these findings, selegiline can improve impaired movements and neuropathic pain caused by SCI (23, 24) and play an essential role in SCI healing (25).

On the other hand, local drug delivery to the epidural zone has grabbed attention recently (26, 27). Despite dura hard tissue, which makes different molecules’ permeability challenging, some molecules could penetrate due to the simple diffusion process because of the osmotic pressure (26, 28). This penetration gets easier when it comes to the drugs that cross the blood-brain barrier and spinal cord readily, such as selegiline (29). For local drug delivery to the spinal lesion site after SCI, hydrogels and scaffolds containing the drug of choice are used so that local drug delivery can be done slowly over days and weeks by implanting hydrogels in the site. Recently, alginate hydrogels have grabbed the attention of drug delivery researchers for this purpose due to their different beneficial properties. Besides the recuperative effects of alginate on SCI (30), the most important alginate characteristics are its biocompatibility, hydrophilicity, simplicity of gelation, and providing an acceptable gradual release of the drug (31). 

**Figure 1:**
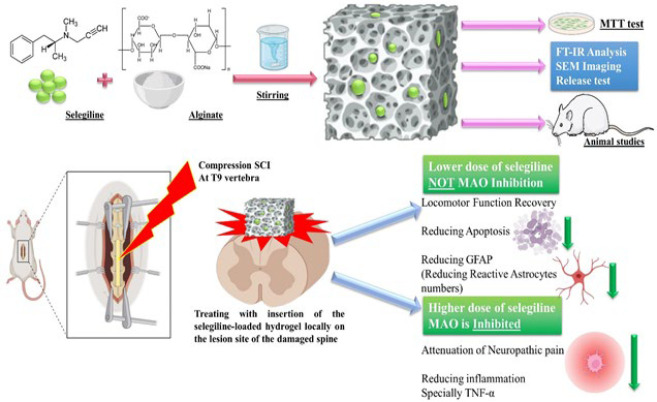
Summary of the process, method, and results of this study

Few reports have investigated selegiline as a medication after SCI. These studies chose the intraperitoneal route for selegiline administration, which makes proper delivery of the drug to the spinal injury site challenging (32-34). This study aimed to investigate the administration of selegiline through a sustained-release alginate hydrogel locally in the spinal lesion site, which could answer the mentioned challenge due to the gradual and local delivery of the drug. The process, method, and results of this study are summarized in Figure 1.

## Materials and Methods

### Materials

Selegiline hydrochloride, the Active Pharmaceutical Ingredient (API), was bought from Zahravi (Tehran, Iran), and cefazolin was obtained from Exir (Tehran, Iran). Fetal bovine serum (FBS), MTT (3-(4,5-dimethylthiazol-2-yl)-2, 5-diphenyltetrazolium bromide), Phosphate buffer saline (PBS), glycerol, penicillin-streptomycin, and dimethyl sulfoxide (DMSO) were purchased from Merck chemicals Co. (Darmstadt, Germany). Moreover, sodium alginate and the other necessary chemicals were purchased from Sigma-Aldrich.

### Animals

This study was performed on 36 male 8-week-old Wistar rats weighing 280±10 g, provided by Tehran University of Medical Sciences (TUMS). The animals were divided randomly into six groups of 6 rats each. The groups were in this order: 1) Sham (healthy control rats that underwent laminectomy without SCI induction), 2) Negative control (SCI-induced rats without any further interventions), 3) Hydrogel group (SCI-induced rats with epidural implantation of alginate hydrogel empty of selegiline), and three other groups which received 2.5, 5, and 10 mg/kg selegiline as treatment. Selegiline was formulated within an alginate hydrogel scaffold and immediately implanted in the epidural spinal lesion site following the induction of SCI. 

SCI models were induced through the following procedure: At the beginning, Ketamine (80–100 mg/kg) and Xylazine (5 mg/kg) were injected intraperitoneally (IP) to induce anesthesia in animals. Subsequently, a complete laminectomy was done at the T9 vertebra, and an aneurysm clip (“YASARGIL® Aneurysm clip system, Titanium mini clips FT712T; closing force, 110 g [1.08 N]; 4.7 mm Blade length; 3.8 mm maximum opening diameter”) was applied at the exposed spinal segment for 60 sec to induce SCI through compression model injury (35, 36). Observation of flutter reflexes of the tail accompanied by abrupt retraction of the hind limbs was considered a sign of proper induction of the SCI model (27). Subsequently, hydrogel specific to each group was implanted in the groups where hydrogel implantation was to be done. Afterward, incisions were sutured, and animals were maintained on a sterile heating pad. Also, necessary postoperative care, such as administration of ringer lactate serum (1 ml per rat), cefazolin (20 mg/kg), and buprenorphine (0.1 mg/kg), were administered for one week after SCI induction. Additionally, since the animals lost the ability to urinate after SCI, we performed manual bladder depletion twice daily until normal function was restored (36). All animal-related procedures of the present work adhered strictly to the guidelines in the “Guide for the Care and Use of Laboratory Animals,” published by the National Academies Press. This study received its ethical approval code from the Shahid Beheshti University of Medical Sciences (SBMU) under the code number IR.SBMU.TEB.POLICE.REC.1402.070. 

### Selegiline-loaded alginate hydrogel preparation

After the preparation of the suitable concentrations of selegiline solution in deionized water, gelation was done by slow addition of alginate to the solution under gentle and uninterrupted stirring. Finally, five drops of 1mM calcium chloride were added to the semi-gel solution to complete the final step of gelation. The final gel contained 4% sodium alginate and the required amount of selegiline according to the determined doses for each group (for the hydrogels of the 2.5, 5, and 10 mg/kg groups, selegiline amount was 4, 8, and 16 mg/ml).

### Cytotoxicity test (MTT assay)

The cytotoxicity test of the different hydrogels was assessed according to ISO 10993-5 protocol evaluated on L929 cells (mouse fibroblast cell line). Initially, L929 cells were cultured in a minimum essential medium (MEM). MEM contains 10% FBS, 50 g/ml streptomycin, and 50 IU/ml penicillin. L929 cells were seeded into 96-well plates in the number of 1×10^4^ cells/well. Cells were incubated overnight. In the next step, hydrogels were sterilized, and a mixture of hydrogels in the medium was prepared with a ratio of 50 mg/ml (hydrogel/medium). Triplicates of each sample were prepared and incubated for 24 hrat 37 °C. For the next step, 100 µl of the extraction of the hydrogels was transferred into each well. The cells were then incubated for 3 days, and an MTT assay was performed. Briefly, MTT solution (0.5 mg/ml in PBS) was added to each well, and for the following four hours, cells were incubated in the standard cell culture condition. The yellow color of MTT was reduced to insoluble purple formazan in the metabolically active cells. The formazan crystals were dissolved in dimethyl sulfoxide (DMSO) while being gently agitated for 30 min. The absorbance was measured at 570 nm for the last step using a microplate reader (Biotek, USA).

### Selegiline-loaded alginate hydrogel characterization

*Fourier-transform infrared (FTIR) spectroscopy*

FTIR spectroscopy (Nexus Por Euro, Bruker, Karlsruhe, Germany) was utilized to analyze the characteristic peaks related to the special bonds in each component and verify the electrostatic interaction between sodium alginate, selegiline, and the final formulation. This method obtains the spectra of samples by the average of 32 scans between 400 cm^−1^ and 4000 cm^−1^ at a resolution of 4 cm^−1^.

### Scanning electron microscopy (SEM)

The alginate hydrogel containing selegiline was air-dried under a fume hood for 24 hr. Next, samples were treated with a thin layer of gold in a vacuum and were subjected to SEM analysis (KYKY-2800; Apparatus Factory, Chinese Academy of Sciences, Beijing, China) at an accelerating voltage of 20 kV.

### In vitro drug release study

To investigate the release profile of selegiline from hydrogels, a total of 1.00 g of the prepared hydrogels was immersed in 4 ml PBS (pH = 7.4) and shacken at 70 rpm at a temperature of 37 °C using a shaker incubator for up to 250 hr. After 2, 4, 8, 12, and 20 hr after incubation, and every 20 hr after that, 500 µl of PBS was replaced with 500 µl fresh one. Finally, release rate of the hydrogels was investigated (27, 37).

### Locomotor scaling

The locomotor activity of the rats’ hind limbs was assessed on the open field for ten minutes using the Basso, Beattie, Bresnahan (BBB) locomotor rating scale (38). This rating scale is from 0 to 21, and scores are based on the number of joints that can move, the ability to bear weight, limb movement ability, and coordination of the joints’ motions. A score of 0 indicates no motor function, whereas a score of 21 denotes complete motor abilities. Each rat was scored various times, including day 0 before the surgery and at 1, 7, 14, 21, and 28 days post-injury (DPI). Each hind leg was scored independently, and the mean was calculated and considered for each rat at each DPI session.

### Neuropathic pain assessment

*Thermal allodynia*

Thermal hypersensitivity was evaluated using a tail-flick test on days 0 (before surgery), 7, 14, 21, and 28 after surgery, and SCI induction following the previously explained method (39). Briefly, rats were held gently, and their tail was put on the tail-flick apparatus (Ugo Basile, Italy). In continuation, a radiant heat stimulus was exposed to the ventral surface of the rat tail about 3-4 cm from the tip of the tail to elicit a tail-flick response. The latency time for the tail withdrawal reflex was recorded in seconds, from the beginning of the heat source activation until tail withdrawal. The test was repeated three times for each rat, and their average was considered their result. A 10-second cutoff limit was considered to avoid harm. 

*Cold allodynia*

Acetone can induce a cold sensation. So, acetone was applied to the hind paws of the rats for 20 sec, and their responses were recorded. A special scoring system was utilized to quantify the withdrawal responses to acetone application (40). Scores of 0, 1, 2, and 3 were assigned based on the rats’ responses, indicating the absence of cold allodynia, mild (scratching of the hind paw), moderate (more intense licking, biting, or withdrawal of the hind paw), or severe (too much licking, multiple paw withdrawals, or even vocalizations) cold allodynia, respectively. The scores of each rat were repeated three times and then summed to obtain a cumulative measure of cold allodynia severity (between 0 and 9) for each time point for each rat.

*Tissue collection*

After 28 days of experiment, a CO_2_ chamber was used to euthanize rats. Subsequently, all of the spinal cords were excised for subsequent investigations. The isolated cord of each rat at the lesion site was then divided into two separate segments. One was kept in a 10% formalin solution (pH: 7.26) for 48 hr for further immunohistochemical studies. The other one, for rapid freezing, was exposed to liquid nitrogen and then stored at -80 °C for later apoptotic factors assessment through western blot examination. 

*TNF- α assay*

On the last day of the experiment, before the euthanasia, 3 ml of blood samples were collected from the tail veins of the rats. TNF-α was assessed in the serum part of the blood using the sandwich enzyme-linked immunosorbent assay (ELISA) according to the protocol provided by the rat TNF-α kit. Briefly, serum samples were obtained by centrifuging the blood samples at 2000 g for ten minutes. For the next step, 100 µl of each serum sample was added to three wells of the pre-coated plate. After that, 50 µl of diluted anti-rat TNF-α secondary antibody was added to each well, and the plate was sealed, followed by incubation at 25 °C for three hours. After several washing steps, the streptavidin-HRP solution was added to each well, and the plate was incubated for 30 min. The final wash was done for the next step, and all wells were incubated with TMB substrate solution for another 15 min. Finally, 100 µl H_2_SO_4_ (stop solution) was used to stop the reaction. The absorbance of each well was measured at 450 nm using a spectrophotometer to determine the optical densities. TNF-α concentrations of each sample were then determined using a standard curve and reported in pg/ml unit.

*Immunohistochemistry and histopathology*

After fixation of each sample in 10% formaldehyde, tissues were embedded in paraffin. Next, transverse and longitudinal sections, each 5 mm thick, were prepared for immunohistochemistry examination. Samples were stained using Rabbit Polyclonal GFAP antibody (ab7260- Abcam).

### Western blot studies

Western blot analysis was utilized to determine how apoptosis-related molecules, including B cell lymphoma/leukemia2 (Bcl 2) and Bcl 2 associated X (Bax), did change because of SCI. Western blot protocol followed the method used in previous studies (41, 42). In brief, the frozen spinal cord segments at the lesion site were homogenized using a freshly prepared lysis buffer containing a complete protease inhibitor cocktail (cOmplete; Roche Diagnostics). After centrifugation, total protein concentrations were assessed using Bradford reagent protein. To separate obtained proteins, 50 µg of total protein was resolved in sodium dodecyl sulfate-polyacrylamide gel electrophoresis (SDS-PAGE 10%) gels and transferred to polyvinylidene difluoride (PVDF) membranes using an electrophoretic transfer system (Roche, Mannheim, Germany). After blocking the membranes with non-fat dry milk, they were incubated overnight at 4 °C with the following antibodies: anti-Bcl-2 (1:200), anti-Bax (1:200), and an anti-β-actin antibody as the loading control (1:200) (all from Santa Cruz Biotech, CA, USA). For visualization, after washing the membranes with PBS and 0.05% Tween-20 (PBS-T), they were treated with a secondary antibody conjugated with horseradish peroxidase (1:5000; BioRad, Hercules, CA, USA) for one hour at room temperature. To visualize protein bands and develop the blots, the BM chemiluminescence detection system (Roche) was utilized, and subsequently, densitometry was performed using the ImageJ software package.

### Statistical analysis

All the independent experiments were carried out in 4 repetitions, and the data were indicated as mean ± standard error of the mean (SEM). The results obtained were analyzed using a one-way analysis of variance (ANOVA) and Tukey’s post hoc test. A P-value below 0.05 was considered statistically significant. 

## Results

### Hydrogel tests

*Cell viability assay*

**Figure 2:**
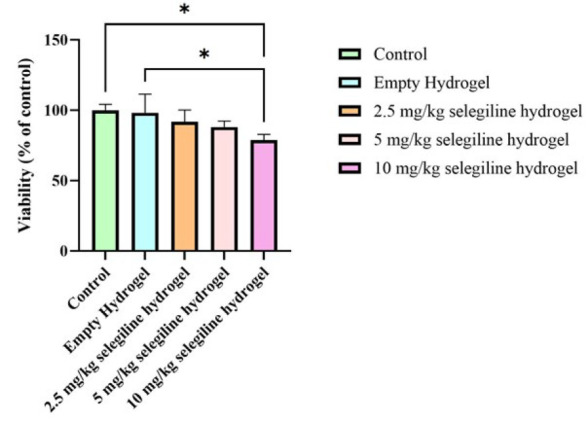
Cell viability of L929 cells after 72 hr of confronting with empty alginate hydrogel and hydrogels containing different doses of selegiline

Comparable viability of L929 cells after confronting with alginate hydrogel and selegiline-loaded alginate with different doses of 2.5, 5, and 10 mg/kg were shown in Figure 2. Tissue culture polystyrene was considered as a negative control group. According to the results, after 72 hr, none of the hydrogels showed any statistically meaningful inhibitory cell growth compared to control groups, but the hydrogel contained 10 mg/kg selegiline. However, even the negative effect of the 10 mg/kg selegiline-loaded hydrogel was very weak statistically (*P*-value=0.0115). Altogether, the results showed that the fabrication processes and the materials had no negative effect on cell viability.

*FT-IR*

**Figure 3:**
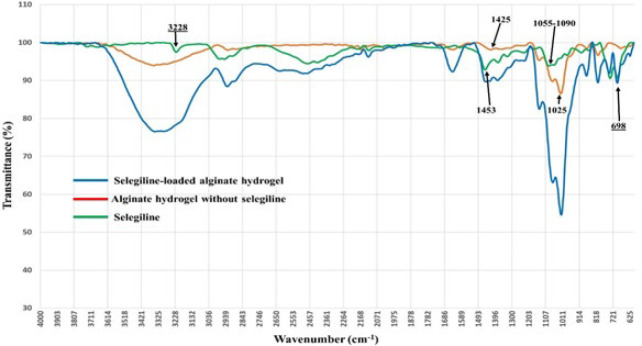
FT-IR spectra of the Sodium alginate, selegiline, and the final formulation

Figure 3 shows the FT-IR spectra of the selegiline-loaded alginate hydrogel besides its constituents. To analyze the spectra in detail, the characteristic peaks of alginate polymer are visible at 1025 cm^-1^, related to the C-O-C stretch, and also at 1640 cm^-1^ and 1425 cm^-1^ wavelengths, which are related to carboxylic acid salt stretch absorptions. Selegiline characteristic peaks can be seen at 698 cm^-1^, related to C≡C bond stretch, and at 1055 cm^-1^ and 1090 cm^-1^, related to -N- stretching. The peak at 1453 cm^-1^ is related to the C-N bond stretch. Moreover, in the 3000 to 3900 cm-¹ range, O-H bond stretching vibrations of both alginate and selegiline are observable, with a characteristic peak at 3228 cm-¹ specific to selegiline (43). Peaks at 2900 to 3000 cm-¹ are due to aliphatic C-H vibrations from both alginate and selegiline. However, many characteristic peaks of alginate hydrogel and selegiline overlap and are amplified sometimes with each other. Although the higher concentration of the polymer makes alginate peaks more prominent, the distinct peaks at 698 cm^-1^, 1090 cm^-1^, and 3228 cm^-1^, related to selegiline specifically, confirm the successful loading (44).

*SEM*

**Figure 4:**
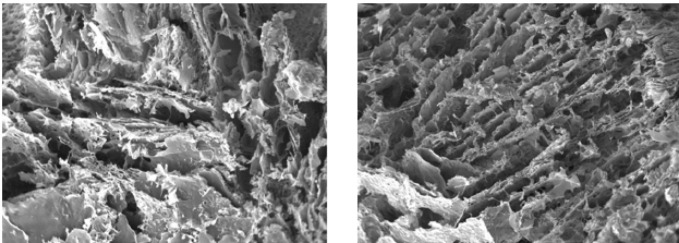
Two images of the microstructure of selegiline-loaded alginate hydrogel captured by scanning electron microscope (SEM)

Figure 4 illustrates SEM images of the inner structure of the freeze-dried selegiline-loaded alginate hydrogel utilized to study its cross-sectional morphology. These SEM images reveal the hydrogels’ highly porous and interconnected pore structure. This characteristic morphology is necessary and beneficial for drug delivery and transportation of nutrients and metabolites. Additionally, this unique morphology provides an excellent environment for cell attachment and proliferation.

*In-vitro drug release analysis*

**Figure 5:**
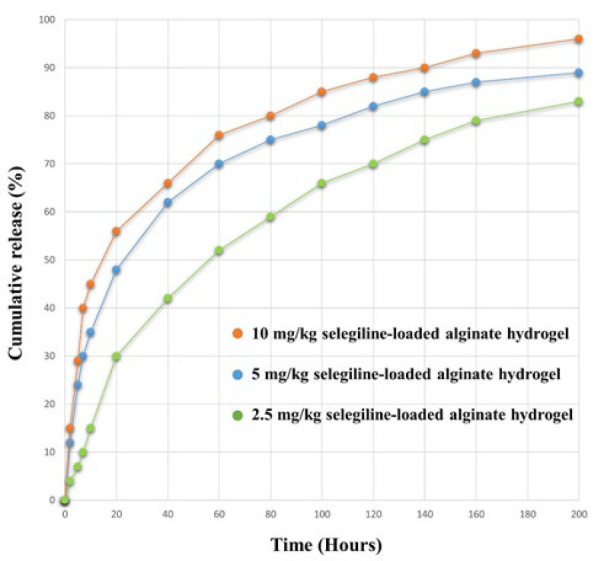
Release profile of different hydrogels

As shown in Figure 5, selegiline was released from the hydrogel slowly and consistently over 200 hr. An initial burst release of selegiline occurred during the first 48 hr, which increased with loading the higher doses of the selegiline due to the hydrophilicity of selegiline (42, 62, and 66% of the loaded drug was released within 48 hr for 2.5, 5, and 10 mg/kg selegiline-loaded alginate hydrogel, respectively). This sustained-release profile, with a maximum release of 83, 89 and 96% for 2.5, 5, and 10 mg/kg selegiline-loaded alginate hydrogel, respectively, indicates that fabrication of the selegiline in the alginate hydrogel was done successfully. 

### Animal studies

*Locomotor activity*

**Figure 6:**
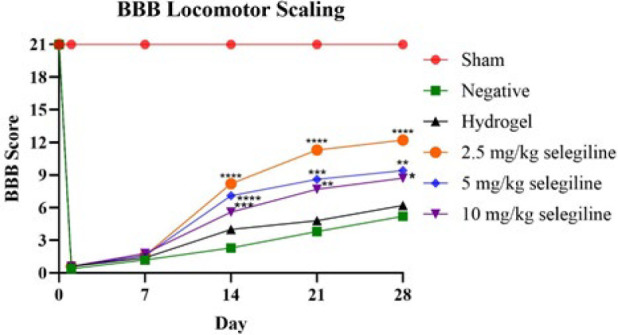
Basso, Beattie, and Bresnahan (BBB) scores of different groups throughout the experiment

Figure 6 shows the BBB score of the different groups during the experiment. Before surgery and SCI induction, all rats were healthy and had a BBB score of 21. After SCI induction, the BBB scores of all groups dropped to zero, while the sham-operated group maintained a score of 21 after surgery. These results confirm successful SCI induction. After 7 days, there were no significant differences between the groups, though mild increases in BBB scores were observed in all groups. In the second week, BBB scores of the groups that received selegiline increased significantly compared with the negative control group (*P*-value≤0.0001). The difference between the groups that received selegiline and the negative control group persisted until the end of the fourth week of the experiment, which indicates the positive effect of selegiline on locomotor impairment recovery. Notably, the empty hydrogel did not affect the BBB scores, indicating that the carrier hydrogel has no beneficial effects. The beneficial effects of selegiline were in an inverse dose manner, and the lowest dose showed the most significant improvement (*P*-value≤0.0001), while higher doses had significant but higher *P*-values.

### Neuropathic pain assessment

*Thermal allodynia*

**Figure 7:**
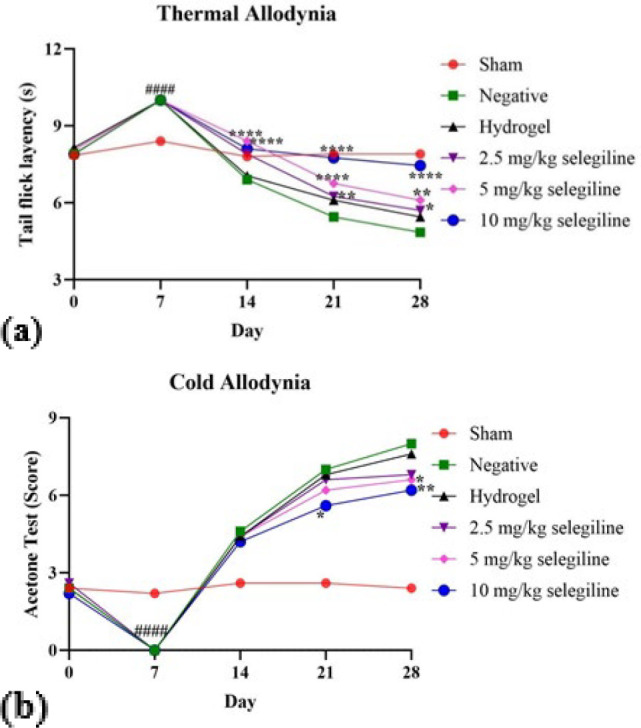
(a) Shows the duration (in seconds) that different rat groups tolerate heat-induced pain on different days of the experiment, and (b) shows the sensitivity of different rat groups to cold-induced pain on different days of the experiment, measured as a score explained in the methods section

Figure 7a shows the time it took for rats of each group to show sensitivity to heat-induced pain throughout the study duration (28 days). The sham group maintained normal sensitivity and showed a consistent tolerance to heat-induced pain throughout the study. On the other hand, other groups’ tolerance changed over time. In the first week post-surgery, all groups showed an insensitivity to heat-induced pain compared to the sham group due to the SCI side effects (*P*-value<0.0001). After the first week, neuropathic pain began to affect heat sensitivity. In the third and fourth week post-injury, SCI-induced groups showed significantly reduced thermal pain threshold compared to the sham group (*P*-value<0.0001), indicating the development of thermal hypersensitivity after SCI as a sign of neuropathic pain. Administration of the selegiline attenuates the SCI-induced thermal hypersensitivity. On the 21st day, different selegiline doses increased heat-induced pain tolerance compared to the negative control group (*P*-value<0.0001 for 5 and 10 mg/kg selegiline and P-value<0.01 2.5 mg/kg). Finally, on the 28th day, it was observed that only administration of the 10 mg/kg selegiline can ameliorate neuropathic pain significantly (*P*-value<0.0001). Also, 2.5 and 5 mg/kg selegiline can ameliorate this issue moderately and significantly (*P*-value<0.1 and *P*-value<0.01, respectively). 

*Cold allodynia*

As Figure 7b shows, the rats in the sham group exhibited normal and consistent sensitivity to the cold allodynia throughout the study duration. In the first week post-surgery, SCI-induced groups displayed insensitivity to the pain compared to the sham group (*P*<0.0001). In the following weeks, due to the development of neuropathic pain, SCI-induced rats show more reaction to the cold allodynia. As with thermal allodynia, the administration of selegiline reduced cold sensitivity in injured rats by the end of the 4th week. Treatment with 5 and 10 mg/kg of selegiline significantly attenuated cold sensitivity (*P*-value<0.1 and *P*-value=0.001, respectively). These results indicate that selegiline can play a role in the amelioration of neuropathic pain caused by SCI. Notably, in these two tests, the empty hydrogel did not affect the sensitivity to the pain, indicating that the carrier hydrogel has no beneficial effects.

*Apoptosis factors*

**Figure 8:**
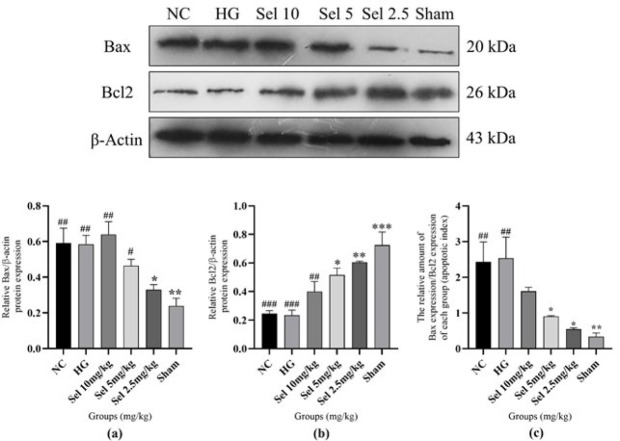
(a) Bax expression in different rat groups, (b) Bcl2 expression in different rat groups, and (c) Bax/Bcl2 ratio (apoptotic index) in different rat groups Groups are labeled below each bar of the charts

The apoptotic mechanism is crucial in SCI research for its role in neuronal cell death, SCI progression, and worsening secondary damage (34, 45). We evaluated Bax and Bcl2 expression by western blot, noting that Bcl2 is anti-apoptotic and Bax is pro-apoptotic (27). The western blot results of different groups are shown in Figure 8. 

To explain the results, the negative control (NC) group, compared to the sham group, showed a considerable increase in Bax molecule expression and a significant decrease in Bcl2 molecule expression (*P*-value = 0.005 and 0.0009, respectively). It can be observed that only the lowest dose of selegiline can reverse the SCI-induced increase in Bax molecule expression (*P*-value = 0.025). Moreover, the western blot results reveal that SCI decreased Bcl2 molecule expression significantly compared to the sham group (*P*-value = 0.0009). Again, lower doses of selegiline were more effective. So, 2.5 and 5 mg/kg doses of selegiline attenuated the SCI-induced decrease in Bcl2 expression to some extent (*P*-value = 0.0043 and 0.0183, respectively). The Bax/Bcl2 ratio was also investigated as an apoptotic index for a more precise assay (the higher index indicated a greater proportion of apoptosis) (27). The index was significantly higher in the negative control group following the SCI than in the sham group (*P*-value = 0.0062). Moreover, doses of 2.5 and 5 mg/kg of selegiline significantly reduced this increased index in the NC group (*P*-value = 0.0104 and 0.0285, respectively). 

Notably, the alginate hydrogel without the drug and the highest dose of selegiline did not change any of these factors compared to the NC group. These findings reveal that alginate hydrogel did not provide any beneficial effects by itself in terms of apoptosis. The ameliorations observed in the groups treated with 2.5 and 5 mg/kg of selegiline were specific to the selegiline. Low doses of the selegiline inhibited apoptosis in the SCI model in an inverse dose-dependent manner.

*Inflammation analysis*

**Figure 9:**
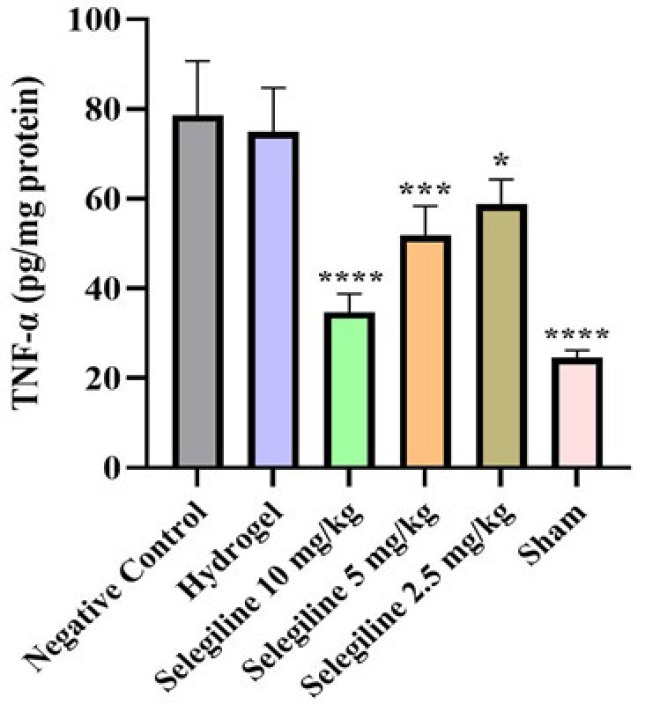
Serum levels of TNF-α in different rat groups, measured by ELISA Groups are labeled below each bar of the chart

Inflammation and inflammatory cytokine production, specifically TNF-α, are crucial in the pathology of SCI (46). Serum levels of the TNF-α of each group are summarized in Figure 9. Based on the results, serum levels of the TNF-α increased in the SCI group compared to the sham group (*P*-value≤0.0001). All three doses of selegiline could significantly reduce the SCI-induced elevated TNF-α back to normal levels (*P*-values for 2.5, 5, and 10 mg/kg selegiline were ≤0.1, ≤0.001, and≤0.0001). Notably, the empty hydrogel did not affect the elevated levels of the TNF-α, indicating that the carrier hydrogel has no beneficial effects. These results reveal a potent antiinflammatory role for selegiline-loaded alginate hydrogel. 

*Immunohistochemical analysis of GFAP*

**Figure 10:**
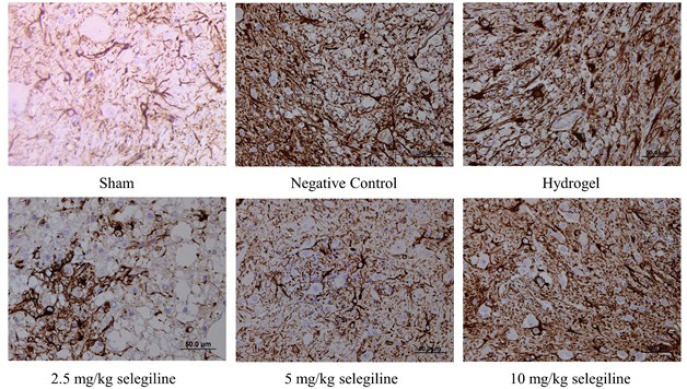
Immunohistochemical analysis of GFAP expression in the lesion site of the spinal tissue in different rat groups. Darker areas indicate higher GFAP expression Groups are labeled below each image. GFAP: Glial fibrillary acidic protein

Glial fibrillary acidic protein (GFAP) is a protein that is found in astrocytes of the CNS uniquely (47). So, the immunohistochemical analysis of GFAP, shown in Figure 10, reveals the number of astrocytes. The negative control group showed a significantly higher number of astrocytes than the sham group due to SCI induction. The empty hydrogel did not ameliorate this, indicating that the carrier hydrogel has no beneficial effects. Moreover, administration of the 2.5 and 5 mg/kg selegiline reduced the number of the astrocytes significantly. However, administering 10 mg/kg selegiline could not change the number of astrocytes. These results indicate that lower doses of selegiline can reduce the number of astrocytes in the tissue and may pose beneficial effects through this mechanism.

## Discussion

Despite the significant prevalence of SCI globally and its highly disabling and costly consequences (5), there are no effective treatment options (11), presenting a substantial ongoing challenge. Many studies have been conducted to find a solution to overcome this challenge. Some studies investigated selegiline as a probable solution due to its neuroprotective effects and its efficacy in treating depression, a common complaint among SCI patients (48-50). A considerable majority of the studies, if not all, reported advantages of the intraperitoneal administration of selegiline in SCI (51). Intraperitoneal administration has limited efficacy because of the fluctuation in the drug concentration and increased likelihood of side effects due to systemic drug administration. To overcome these obstacles, recent studies investigate different implantable sustained-release hydrogels as potential SCI treatment options (37, 52-54). Among various kinds of hydrogels, alginate scaffolds have been reported to promote axonal regeneration to some extent after SCI. Also, their unique characteristic and shape make them a good choice for local drug delivery, providing a suitable environment for cell attachment and proliferation (27, 55).

Considering mentioned challenges besides the benefits of selegiline and alginate, we made an effort to integrate different doses of selegiline into an alginate hydrogel scaffold to: first, ensure the controlled and sustained-release profile of the selegiline; second, reduce the potential risks associated with its systemic administration; and finally, synergize the alleviating effects of the selegiline with alginate hydrogel to mitigate secondary injury mechanisms, especially apoptosis. Our findings shed light on several key aspects, including the biocompatibility of the hydrogel, drug release kinetics, and the therapeutic efficacy of selegiline in improving locomotor function, alleviating neuropathic pain, and its beneficial effects at the molecular levels in a rat SCI model. Biocompatibility is the first property that must be checked in safe and efficient biomaterial or implantable hydrogels, making them suitable for drug delivery applications (56). One of the best methods, the preferred approach to evaluate biocompatibility, is MTT (56). Previously, alginate was used in local spinal cord drug delivery due to its outstanding biocompatibility and some other properties (57). Our results from the cell viability assay also emphasized its biocompatibility. As the second priority, the property of sustained drug release must be assessed. Different studies showed a suitable sustain-release potential for alginate hydrogels for 6 to 8 days (58, 59). Our findings indicate effective encapsulation and gradual release of selegiline. Following previous studies, we demonstrate a sustained-release profile of selegiline from the hydrogel over 200 hr, except for the initial burst release. Encapsulation of hydrophilic leads to in water absorption and faster drug release (60). As a result, encapsulation of higher doses of selegiline as a hydrophilic drug results in more significant burst release. These findings support the feasibility of using the alginate hydrogel as a delivery vehicle for selegiline in SCI treatment. Following the encapsulation, SEM analysis is necessary to investigate the suitability of the hydrogel structure. The hydrogel should have a unique characteristic shape with a highly porous and interconnected pore structure to provide a suitable environment and nutrients for cell attachment and proliferation (27). Also, the absence of the crystalized form of the loaded drug is another critical factor in the optimum hydrogel formulation, which makes the release profile more consistent and sustained (61). The accordance of the SEM image of our hydrogel with the standard critics indicated in previous studies emphasizes its suitability.

Our animal studies showed that selegiline leads to significant improvements in locomotor function and alleviates neuropathic thermal and cold allodynia in rats with SCI in a dose-dependent manner. These findings are consistent with previous studies, which reported similar benefits of selegiline (23, 33, 34). However, those studies face limitations, including intraperitoneal administration and investigating only a single selegiline dosage without considering a range of dosages from minimum to maximum. Investigating the effects of the different doses of selegiline has resulted in finding an important point. Previous studies indicated that higher doses of selegiline result in greater inhibition of MAO enzymes (62). So, selegiline dose-dependent nociceptive effects indicate the role of MAO inhibition in SCI neuropathic pain attenuation. Furthermore, a very recent study shows that selegiline poses antiinflammatory effects dose-dependently. Although the precise mechanism requires further investigation, the observed dose dependency suggests that the inhibition of MAO plays a crucial role in selegiline antiinflammatory effects. This study even demonstrates the potential of repurposing selegiline to treat rheumatoid arthritis. This study suggests reduced hydrogen peroxide (H_2_O_2_) generation and inhibition of pro-inflammatory cytokines occur through decreased catecholamine breakdown (63). In line with previous studies, we observed that selegiline treatment resulted in a significantly reduced expression of TNF-α in a dose-dependent manner. This observation, alongside a significant elevation of the TNF-α concentration following the induction of SCI, indicates its antiinflammatory properties through the probable mentioned mechanism. Conversely, the anti-apoptotic effects of selegiline showed alleviation in an inverse dose-dependent manner. Specifically, previous research has shown that, at high concentrations, selegiline induces apoptosis in several neurodegenerative disorders due to its dopaminergic effects and free radical generation (64). Meanwhile, lower concentrations mitigate apoptosis by reducing adverse effects and preventing neuronal death (65). These observations explain and align with our results, which demonstrate that selegiline exhibited anti-apoptotic in an inverse dose-dependent manner. The inverse dose-dependent manner of motor function improvement, similar to that observed in apoptosis, brings to mind that the regulation of apoptosis is more critical than the MAO inhibition and antiinflammatory effects in locomotor function recovery. Our results indicate that lower dosages result in better outcomes and highlight different dosage-related effects, which helps with further investigations. Additional research is needed to determine the optimum dosage for selegiline and other MAOIs. Moreover, many other critical factors crucial for SCI alleviation should be assessed, and their correlation with MAO inhibition needs to be clarified.

GFAP is a protein found uniquely in astrocytes within the CNS (47). Evaluating spinal GFAP expression through immunohistochemistry indicates the number of reactive astrocytes present. Evaluating spinal GFAP expression through immunohistochemistry shows the number of reactive astrocytes present. Until very recently, astrocytes were believed to be detrimental to axon regeneration. This belief leads to efforts targeting the inhibition of GFAP expression for SCI treatment (66, 67). Recently, there have been some studies that have challenged this view. Notably, a recent important study indicates that there is not any direct correlation between astrocyte presence and glial scar formation. Contrarily, it suggests that the presence of astrocytes up to some ratio can even support the regeneration of axons (68). Another study corroborated this idea and demonstrated that axon regrowth occurred only in the presence of astrocytes (69). Our results show a dose-dependent increase in astrocyte presence with higher selegiline dosages. As we explained above, MAO inhibition occurs at higher doses, and this dose-dependency suggests a role for MAO inhibition in increasing astrocyte presence. Next, functional recovery and apoptosis were severely impaired at the highest dosage, associated with the greatest presence of astrocytes. Conversely, lower dosages, which showed better recovery outcomes, resulted in the presence of astrocytes, but in limited numbers. The precise extent to which astrocytes are beneficial remains unclear. Due to those new controversial findings and our results, further investigation is required to clarify the beneficial or detrimental roles of astrocytes, determine the extent of their presence at which they are harmful or beneficial, assess the effect of MAO inhibition on astrocytes presence, and elucidate the relationship between astrocytes and attenuation outcomes.

## Conclusion

Selegiline-loaded alginate hydrogel was successfully fabricated using the cross-linking gelation method. The hydrogel structure was highly porous and interconnected, which provided a suitable environment for neuron regeneration. Moreover, our findings suggested that low doses of selegiline can recover impaired locomotor function, inhibit apoptosis, and regulate the number of astrocytes. On the other hand, higher doses showed antiinflammatory and neuropathic pain reliever effects. These findings confirmed the hypothesis that sustained-release selegiline-loaded alginate hydrogel is effective at alleviation of SCI if locally implanted. Notably, further investigations are warranted to assess the beneficial effects of selegiline in combination with different scaffolds, explore more different doses of selegiline and determine the best dose, determine astrocytes role in SCI more precisely, and clinically assess selegiline in SCI.

*weak statistical difference with a *P*-value of 0.0115.

showing a porous structure suitable for spinal implantation and sustained drug release.

orange line denotes 10 mg/kg selegiline-loaded alginate hydrogel, blue denotes 5 mg/kg selegiline-loaded alginate hydrogel, and green denotes 2.5 mg/kg selegiline-loaded alginate hydrogel

**** indicates a statistically significant difference with a *P*-value ≤ 0.0001. *, **, and *** indicate moderately statistically significant differences with *P*-values of <0.1, <0.01, and <0.001, respectively.

Different groups are represented using distinct colors beside the charts.

Significant differences with the sham group are indicated by #### (*P*-value<0.0001), and with the negative control group by **** (*P*-value<0.0001), *** (*P*-value<0.001), ** (*P*-value<0.01), and * (*P*-value<0.1), respectively.

*, **, and *** show significant differences compared to the negative group with *P*-values of <0.05, <0.01, and <0.001, respectively. The #, ##, and ### show significant differences compared to the sham group with *P*-values of <0.05, <0.01, and <0.001, respectively. NC stands as Negative control group, HG as Hydrogel group, and Sel as selegiline.

The results are reported in pg/mg protein. **** indicates a statistically significant difference with the negative control group with a *P*-value ≤0.0001. *, **, and *** indicate moderately statistically significant differences with the negative control group with *P*-values of <0.1, <0.01, and <0.001, respectively.

## Data Availability

Data will be available upon reasonable request.

## Declaration

We have not used any AI tools or technologies to prepare this manuscript.
